# Microsatellite Instability in the Tumor Microenvironment: The Role of Inflammation and the Microbiome

**DOI:** 10.1002/cam4.70603

**Published:** 2025-04-15

**Authors:** Elizabeth Vargas‐Castellanos, Andrés Rincón‐Riveros

**Affiliations:** ^1^ Hospital Universitario Mayor‐Mederi Universidad del Rosario Bogotá Colombia; ^2^ Facultad de Ciencias de la Salud Universidad Colegio Mayor de Cundinamarca Bogotá Colombia

**Keywords:** carcinogenesis, inflammation, microbiome, microsatellites, mismatch repair

## Abstract

**Background:**

Microsatellite instability (MSI) is a hallmark of DNA mismatch repair (MMR) deficiency that leads to genomic instability and increased cancer risk. The tumor microenvironment (TME) significantly influences MSI‐driven tumorigenesis, and emerging evidence points to a critical role of the microbiome in shaping this complex interplay.

**Methods:**

This review comprehensively examines the existing literature on the intricate relationship between MSI, microbiome, and cancer development, with a particular focus on the impact of microbial dysbiosis on the TME.

**Results:**

MSI‐high tumors exhibited increased immune cell infiltration owing to the generation of neoantigens. However, immune evasion mechanisms such as PD‐1/CTLA‐4 upregulation limit the efficacy of immune checkpoint inhibitors (ICIs) in a subset of patients. Pathobionts, such as Fusobacterium nucleatum and Bacteroides fragilis, contribute to MSI through the production of genotoxins, further promoting inflammation and oxidative stress within the TME.

**Conclusions:**

The microbiome profoundly affects MSI‐driven tumorigenesis. Modulation of the gut microbiota through interventions such as fecal microbiota transplantation, probiotics, and dietary changes holds promise for improving ICI response rates. Further research into cancer pharmacomicrobiomics, investigating the interplay between microbial metabolites and anticancer therapies, is crucial for developing personalized treatment strategies.

## Graphical abstract

The microbiome, an environmental factor, can contribute to DNA damage and affect mutation accumulation. Infections with genotoxin‐producing pathobionts can induce inflammation and oxidative stress through reactive nitrogen and oxygen species, which may influence MSI.

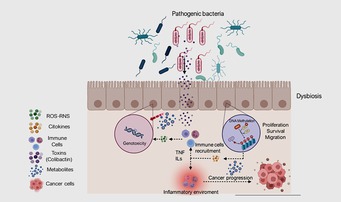

**Abbreviations**
CAFs: cancer‐associated fibroblasts
CRC: colorectal cancer
dMMR: deficiency in the MMR system
EMAST: elevated microsatellite alterations at selected tetranucleotides
EPEC: enteropathogenic *E. coli*

ETBF: enterotoxigenic *Bacteroides fragilis*

HNPCC: hereditary nonpolyposis colon cancer
IARC: Agency for Research on Cancer
ICIs: immune checkpoint inhibitors
LAB: lactic acid bacteria
MMR: mismatch repair system
MS: microsatellites
MSI: microsatellite instability
RNS: reactive nitrogen species
ROS: reactive oxygen species
STRs: short tandem repeats
TCGA: The Cancer Genome Atlas Project
TME: tumor microenvironment

## 1 Introduction

Cancer, a complex group of diseases characterized by disruption of cell proliferation, differentiation, and resistance to cell death, evolves through a process known as carcinogenesis. During this process, a series of mutations gradually accumulate in genes that regulate these cellular functions, ultimately transforming tumor cells and conferring them significant advantages in proliferation, invasion, and immune system evasion [1].

The accumulation of mutations is a hallmark of cancer development, leading to various alterations such as deficiencies in DNA repair systems, activation of proto‐oncogenes, and loss of tumor suppressor gene function. Environmental factors can contribute to DNA damage, further complicating the multifaceted nature of this disease [2]. Although some determinants of cancer development are known, much remains to be discovered. Recent advances in experimental techniques have shed light on tumor heterogeneity and its microenvironment, revealing the mechanisms that influence mutation accumulation and cancer predisposition [3].

Over the past few decades, our understanding of the impact of the microbiome on tumor development and its potential to influence therapeutic outcomes has expanded in modern oncology. Previously, the microbiota–cancer relationship primarily involved pathogenic factors. However, advances in metagenomics and metatranscriptomics have allowed researchers to associate the microbiome (including genes, toxins, and metabolites) with various hallmarks of cancer, making it a significant area of interest in cancer biology [4, 5].

This review focuses on microsatellite instability (MSI), a key process in tumor development, with special emphasis on factors within the tumor microenvironment (TME) that affect the mismatch repair (MMR) system, a well‐known player in this process.

In this review, we explored the link between inflammation associated with the microbiome and its influence on MSI. We will also explore the impact of infection with genotoxin‐producing pathobionts and their role as inducers of inflammation and oxidative stress through reactive nitrogen species (NOS) and reactive oxygen species (ROS). This manuscript aims to underscore the importance of comprehensive research, diagnosis, and treatment of cancer to address the microbiome–cancer axis. By doing so, we can enhance decision‐making that benefits patients and positively impacts individual and population management.

## 2 MSI and Microbiome Interplay in Cancer Development and Therapy

MSI and deficiencies in the mismatch repair (dMMR) system are significant factors in the development of several cancers, notably colorectal and endometrial cancers. MSI results from the accumulation of mutations in short tandem DNA repeats (microsatellites) due to errors during replication, leading to genomic instability. MSI is particularly prevalent in Lynch syndrome–associated and sporadic tumors, where it drives tumorigenesis by generating neoantigens that stimulate an immune response [6, 7].

**FIGURE 1:**
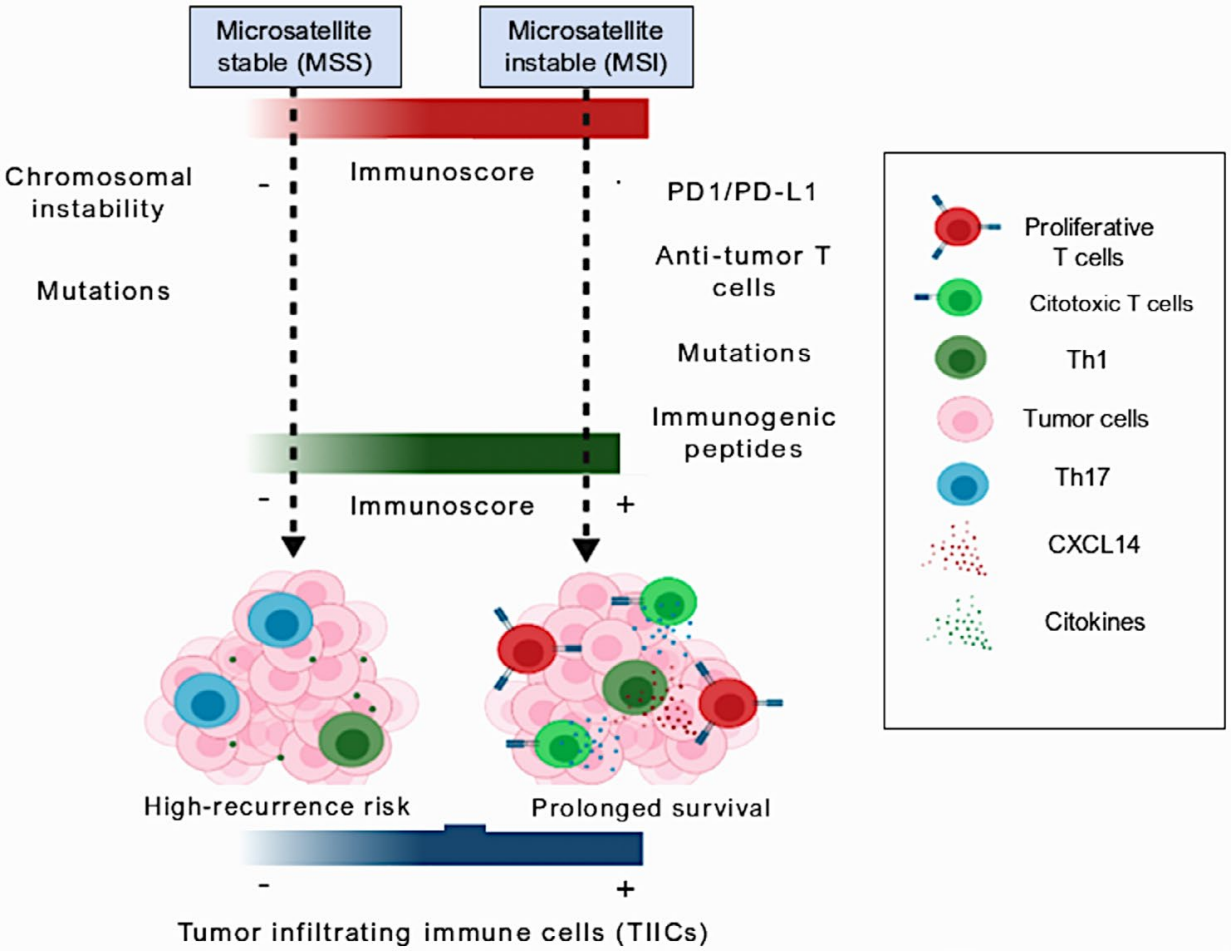
In colon cancer, microsatellite instability is a favorable predictive factor for increased neoantigen production by tumors, which favors an antitumor immune response.

The immunogenic potential of MSI tumors often results in a favorable prognosis, with high levels of tumor‐infiltrating lymphocytes and increased sensitivity to immune checkpoint inhibitors (ICIs), particularly in MSI‐high (MSI‐H) cancers. Tumors with MSI‐H/dMMR status exhibit heightened infiltration of immune cells, specifically T lymphocytes, which engage in antitumor responses [8] (Figure 1). However, tumor cells often counteract this response by upregulating immune checkpoint molecules such as PD‐1 and CTLA‐4, leading to an immunosuppressive TME. ICIs targeting these checkpoints have shown promise, particularly in MSI‐H tumors, due to their high mutational load and resultant neoantigen presentation [9, 10].

Nevertheless, some MSI‐H tumors still exhibited resistance to immunotherapy, suggesting the presence of additional immunosuppressive pathways that require further exploration. The microbiome plays an emerging role in the modulation of immune responses in MSI‐associated cancers. In particular, the gut microbiome can influence immune cell activation, tumor immunogenicity, and response to ICIs. Specific bacterial species have been linked to improved ICI responses, whereas others may promote resistance by fostering an immunosuppressive environment.

Interventions targeting the microbiome, such as fecal microbiota transplantation (FMT) or probiotics, have shown potential as adjuncts to enhance therapeutic outcomes in MSI‐H cancer. Next‐generation sequencing and liquid biopsies are advancing our understanding of MSI‐related carcinogenesis by uncovering biomarkers and therapeutic targets, such as kinase fusions treatable with kinase inhibitors. Personalized immunotherapies, including neoantigen vaccines, are promising for MSI‐stable tumors and are expanding the treatment options for MSI‐H cancers [11, 12].

Future research should focus on optimizing microbiome‐targeted therapies and further characterizing MSI‐associated immune responses to refine predictive biomarkers and overcome resistance mechanisms in cancer therapies.

### 2.1 Mismatch Repair System

DNA is continuously stressed by exposure to environmental or exogenous factors, such as chemical or physical agents (smoking, asbestos, and radiation), as well as endogenous agents, such as ROS and NOS. Another source of DNA sequence variation occurs during the normal physiological processes of replication and repair through the erroneous inclusion of nucleotides. These errors are inherent in the performance of the DNA polymerase [13].

To avoid deleterious damage and safeguard the integrity of the genome, there are different DNA surveillance and repair mechanisms, one of which is MMR. This system comprises a group of genes that encode a conserved protein system in mammals and humans. MMR has its origin in studies using *Escherichia coli* and its repair genes *MutS* and *MutL* [14].

**FIGURE 2:**
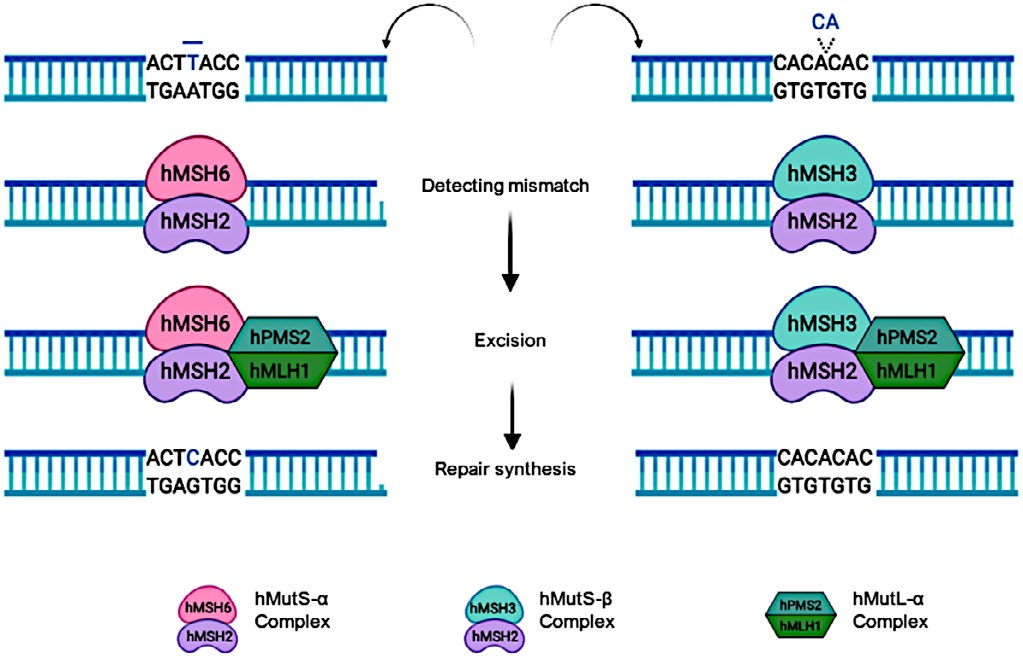
The mismatch repair system and its heterodimer conformation in response to base unpairing or nucleotide insertion or deletion.

In humans, the genes involved in the MMR system are referred to as MSH and MutS homologs (MutL homologs), and this system consists of three main steps: lesion recognition, repair initiation, lesion excision, and DNA resynthesis. The specificity of the MMR system is primarily due to the correction of missing bases and the insertion and deletion of bases during DNA replication and recombination (Figure 2). Proteins of the MMR system must act as heterodimers, and eight genes are involved in this system: MSH genes (*hMSH2*, *hMSH3*, *hMSH2*, *hMSH5*, and *MSH6*) and *MLH* genes (*hMLH1*, *hPMS1*, *hMLH2*, *hMLH3*, *hPMS2*, or *hMLH4*) [15, 16].

In eukaryotic cells, two heterodimers form the MMR system. The first is the correction protein hMSH2, which can form two different heterodimers for the correction of missing bases, one with hMSH6 and the other with hMSH3, known as MutSα and MutSβ, respectively. The hMSH2–hMSH6 complex detects single‐base disappearance and dinucleotide insertions or deletions, whereas hMSH2–hMSH3 identifies loops formed from insertions and deletions of up to 13 nucleotides [17]. MutL homologs found in humans are part of the GHKL (giarase/HSP90/histidine kinase/MutL) family of proteins and can generate a ring around the DNA helix. The hMLH1 protein can form heterodimers with three different types of monomers, postmeiotic segregation enhanced (PSM), MLH2, and MLH3, which are recruited after the detection of base unpairing by MutSα or MutSβ [18] (Figure 2).

## 3 Tumor Microenvironment

The genomic behavior of tumor cells is greatly influenced by their environment, which is known as the TME. Studies have shown that the number of mutations in tumor cells can be increased up to fivefold in 3D models compared to simple cell cultures [19]. The TME is composed of different cell types, including macrophages, cancer‐associated fibroblasts (CAFs), endothelial cells, pericytes, T lymphocytes, NK cells, mesenchymal stem cells, myeloid‐derived suppressor cells, and tumor cells [20].

### 3.1 Inflammation

#### 3.1.1 Inflammation in the TME and MSI

Another alteration in microsatellites is elevated microsatellite alterations at selected tetranucleotides (EMAST) loci, modifications already documented in colorectal cancer (CRC) and other solid tumors. In CRC, it has been established that inflammatory factors produced in the TME are associated with low microsatellite instability (MSI‐L) and EMAST, preferably in ulcerated tumors with oxidative stress, interleukin 6 (IL‐6) and prostaglandin E2 (PGE2) production, leading to the displacement of MSH3 from the nucleus to the cytoplasm and increasing the appearance of EMAST. Treatment of CRC cells with IL‐6 has also been shown to induce EMAST in vitro [21, 22].

In addition to hypoxia, the TME contributes to other stimulating factors of MSI and ROS, produced by inflammatory cells and the TME microbiota, which affect not only developing tumor cells but also stromal cells, generating oxidative stress that favors tumor development and an increase in hypermutated phenotypes [23].

The microbiome refers to the collection of genetic material and products of all microorganisms (bacteria, fungi, protozoa, viruses, and archaea) that live symbiotically within the human body. Recent advancements in technology have allowed for a deeper understanding of the microbiome's role in cancer, specifically in terms of genomic instability [24]. Imbalances in the microbiome, referred to as dysbiosis, can disrupt the immune system, cause tissue damage, and lead to inflammation. These imbalances have been linked to an increased risk of cancer [25].

Inflammation can create a tumor‐promoting microenvironment, which influences each stage of tumorigenesis, inducing DNA damage and altering the cellular mechanism of DNA repair; the release of ROS by macrophages in response to inflammatory cytokines directly induces DNA rupture, causing mutations and stimulating transcription factor pathways (NRF2, NF‐κB) that alter cell growth to produce cancer [26].

## 4 Microbiome and Genomic Instability

The study of the intratumoral microbiome is transforming our understanding of cancer by revealing how specific microbes contribute to tumor initiation, growth, and spread. Microorganisms within tumors originate from various sources: damaged mucosal barriers, nearby tissues, or the bloodstream—each introducing unique microbial communities that shape cancer progression. These microbes influence the TME, affecting immune responses, promoting inflammation, and potentially fueling genetic instability [27]. Recognizing the diverse origins and roles of intratumoral microbes underscores their potential as therapeutic targets, advancing early cancer detection and enhancing treatment strategies tailored to the microbiome's impact on cancer dynamics.

### 4.1 CRC Models With MSI‐Microbiome Deregulation

CRC is a prevalent disease worldwide and is the third most common cancer. In Colombia, it is the third most common cancer, with approximately 11,200 cases and 5600 deaths recorded in 2022 [28]. The intestinal microbiota is a complex ecosystem home to more than 10^11^ microorganisms, with a significant presence in the colon [29]. This ecosystem is closely associated with both health and disease, with some microorganisms being recognized for promoting carcinogenesis, such as *Helicobacter pylori* in gastric cancer (GC) and *Salmonella Typhi* in bile duct cancer [30, 31, 32].

**FIGURE 3:**
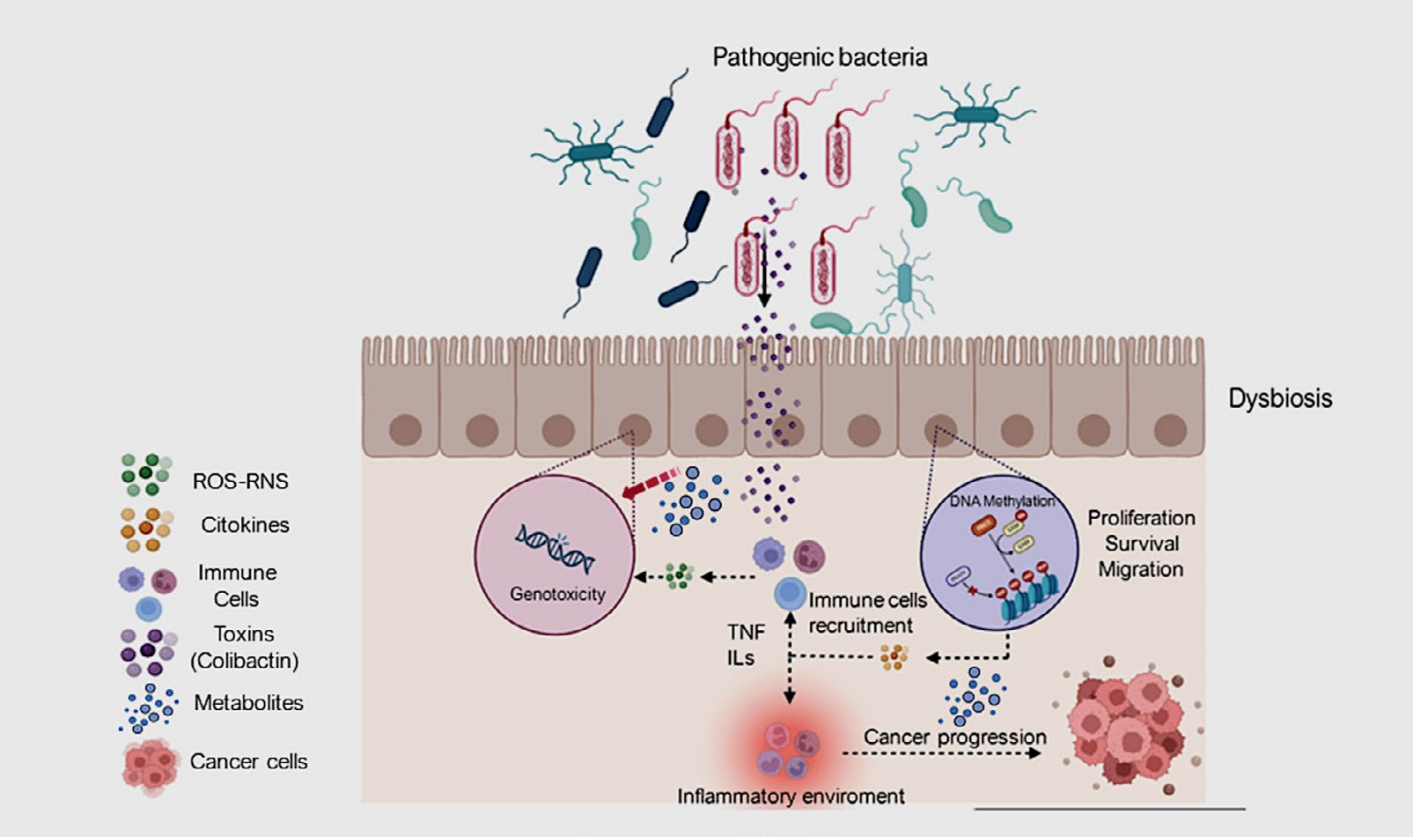
Dysbiosis of the microbiome in colorectal cancer increases colonization by pathogenic bacteria that stimulate the production of genotoxic agents and metabolites that induce DNA damage, inflammation, and carcinogenesis.

Research in murine models, preclinical studies, and human reports has provided evidence of the impact of dysbiosis on the development of CRC. This is characterized by differential bacterial diversity between the tumor tissue and adjacent normal mucosa. The induction of proinflammatory cytokines, generation of ROS, and action of genotoxins are some of the mechanisms associated with the carcinogenesis of certain bacteria, such as *Bacteroides fragilis* , *Fusobacterium nucleatum* , and *E. coli* [24] (Figure 3).

*Bacteroides fragilis* is a gram‐negative anaerobe comprising about 25% of the gut microbiota and exists in two forms: enterotoxigenic *B. fragilis* (ETBF) and non‐toxigenic *B. fragilis* (NTBF). ETBF produces the *B. fragilis* toxin (BFT), which induces chronic intestinal inflammation and tissue damage, thereby playing a significant role in CRC pathogenesis. This toxin disrupts the epithelial barrier and activates pro‐inflammatory pathways, particularly NF‐κB, fostering an environment conducive to tumorigenesis [33].

*F. nucleatum* is an anaerobic gram‐negative, non‐spore‐producing bacillus that is part of the oral microbiota and has been associated with a spectrum of infections, making it a potential oncogene. In recent studies, such as the ColoCare Study (NCT02328677), *F. nucleatum* has been linked to colon cancer carcinogenesis, chemoresistance, metastasis, and poor prognosis [34, 35]. Moreover, other studies, including NCT03698461, NCT04148378, NCT05635149, and NCT03941080 (https://ClinicalTrials.gov/ 06 November 2024), have incorporated the study of the microbiome as a potential determinant in carcinogenesis and the success of antitumor therapy in colon cancer.

From a public health perspective, an evaluation of different populations to determine the presence of *F. nucleatum* is necessary to validate its role as a possible diagnostic and prognostic marker, as studies have shown that *F. nucleatum* has implications for therapeutic success in patients with CRC. *F. nucleatum* promotes resistance to chemotherapy and metastasis by activating TLR4, MyD88, CARD3, and some miRNAs in the autophagy pathway [36, 37].

Analysis of The Cancer Genome Atlas (TCGA) data showed higher mutation burdens and unfavorable molecular features, such as MSI and CpG island methylator phenotype (CIMP), in CRC cases with elevated *F. nucleatum* levels. Experiments in cell cultures and mice indicated that *F. nucleatum* induces DNA double‐strand breaks, marked by increased γ‐H2AX, and disrupts DNA repair mechanisms by downregulating FOXO3 and BAX [38, 39].

*F. nucleatum* preferentially colonizes KRAS p.G12D mutant CRC tumors. *F. nucleatum* binds to the DHX15 protein on CRC cells, activating the ERK/STAT3 signaling pathway, which promotes tumor growth. Knocking out Dhx15 in mice with KRAS p.G12D mutant CRC reduces tumor progression. Additionally, *Parabacteroides distasonis* competes with *F. nucleatum* for DHX15 binding, and oral administration of *P. distasonis* in mice with KRAS p.G12D mutant CRC reduces tumor growth and *F. nucleatum* levels [40]. *F. nucleatum* clade 2 is a dominant species within CRC tissues, indicating its critical role in fostering a pro‐tumorigenic environment. This clade's prevalence is associated with a distinct microbial composition that differentiates cancerous from noncancerous tissues. Furthermore, *F. nucleatum* clade 2 appears to promote inflammation and immune evasion in the TME by interacting with host immune cells, potentially skewing immune responses to favor tumor progression [41].

The possibility of evaluating the presence of *F. nucleatum* in feces or in tumor tissue would be a useful tool for decision‐making, considering that the presence of the bacterium is closely associated with CRC and other tumors, and that in murine and human models, antimicrobial therapy against *F. nucleatum* has been evaluated, resulting in a reduction in tumor growth [42, 43].

Another bacterium associated with colon cancer carcinogenesis is *E. coli* , a strain that is pathogenic and produces genotoxins. Some in vitro and in vivo studies have associated the production of colibactin, a secondary metabolite toxin of the polyketide synthase (pks+) enzyme complex, with the deregulation of MLH1 caused by the induction of oxidative stress in infected cells [44].

The secretion of the EspF protein in enteropathogenic *E. coli* (EPEC) also induces the production of ROS and stimulates posttranscriptional changes in the genes of the MMR system. These findings are supported by recent results showing that in patients with colon cancer, there is a shift from commensal to pathogenic *E. coli* populations, leading to a decrease in the phylogenetic diversity of the intestinal microbiota [45, 46].

In a recent study by Pleguezuelos‐Manzano et al., the ability of pks+ to induce a mutational signature in intestinal organoids was described. Colibactin generates double‐stranded DNA breaks (SBSs) and insertions and deletions (indels). These findings, along with those of Stratton et al., confirm the oncogenic potential of genotoxic strains of *E. coli* in healthy carriers, increasing the risk of developing CRC [47, 48].

### 4.2 Models for GC: Dysregulation of the MSI Microbiome

GC is one of the most lethal neoplasms worldwide. According to the latest GLOBOCAN report, it accounted for more than 660,000 fatalities, with an estimated incidence of nearly one million new cases in 2022. In Colombia, as reported by Cancer Today, in 2022, there were approximately 8938 new cases of GC and a staggering mortality rate of approximately 6901 deaths attributed to this form of cancer, confirming its status as the deadliest cancer in the country [28].

Abate et al. investigated the role of the microbiome in GC by analyzing microbial profiles across two large, diverse patient cohorts: the Memorial Sloan Kettering Cancer Center (MSKCC) and TCGA. The analysis revealed significant reductions in microbial diversity in GC tissues compared to adjacent nonmalignant samples (*p* < 0.05). Notable bacterial enrichments included *Helicobacter*, *Lactobacillus*, *Streptococcus*, *Prevotella*, and *Bacteroides*, with distinct microbial profiles associated with different GC molecular subtypes, particularly in microsatellite instability‐high (MSI‐H) tumors [49].

#### 4.2.1 Helicobacter pylori

*H. pylori* is a gram‐negative bacterium classified as an oncobacterium capable of colonizing and persisting within the human stomach. Infection with this bacterium is recognized as the most prevalent chronic infection globally, with a prevalence rate of up to 75% [50].

The MMR system, which is crucial for correcting base mismatches and preventing MSI [51], appears to be compromised in *H. pylori* infection. Recent findings indicate that *H. pylori* infection downregulates 14 of 23 MMR‐related genes, affecting key components, such as MSH2 and MSH6, which are responsible for damage recognition, and MLH1, which is involved in nick insertion, in a CagA‐independent manner [52]. Supporting prior research, MLH1 silencing may occur through promoter hypermethylation, whereas MSH2 and MSH3 are downregulated through miR‐155‐5p and miR‐3163, respectively [53]. Additionally, downregulation of accessory factors, such as PCNA and RFC, which support MutS and MutL activities, suggests potential impairment in DNA damage recognition, synthesis, and ligation. Moreover, reduced levels of replication protein A (RPA), DNA polymerases (POLD1‐3), and ligase 1 (LIG1), which are CagA‐dependent, may exacerbate replication stress and genomic instability [52].

Studies have revealed a complex and multifaceted role of *H. pylori* in advancing immune evasion and GC progression through diverse convergent mechanisms centered on PD‐L1 upregulation. Evidence indicates that *H. pylori* virulence factors not only enhance PD‐L1 expression directly within tumor cells but also influence surrounding stromal elements, such as lymphatic endothelial cells, thereby creating an immune‐evasive TME that promotes GC growth [54].

This modulation occurs via cellular and extracellular pathways, including microRNA‐loaded small extracellular vesicles and PD‐L1‐enriched exosomes, both of which suppress T‐cell activity and facilitate immune escape. Furthermore, the activation of signaling cascades such as PI3K/Akt, JAK/STAT, and MAPK underscores the mechanistic link between pathogen‐derived factors and immune checkpoint regulation [55].

Together, these pathways illuminate a sophisticated interplay between infection and tumor immune escape, emphasizing the therapeutic potential of targeting PD‐L1 with ICIs to counteract infection‐induced immune suppression and limit GC progression in *H. pylori* ‐associated cases [56].

Regarding the modulatory role of bacteria in GC therapy, findings indicate that *H. pylori* upregulates PD‐L1 expression, which diminishes the effectiveness of tumor immunotherapy. Activation of the Sonic Hedgehog (SHH) pathway by *H. pylori* infection promotes both PD‐L1 expression and tumor cell proliferation in GC, contributing to resistance against immunotherapy [57].

Methylation of the promoter region of GLI1, a crucial transcription factor in the SHH pathway, is reduced by *H. pylori* in an m6A‐dependent manner, leading to increased GLI1 expression and enhanced tumor proliferation. Furthermore, *H. pylori* upregulates PD‐L1 expression in various cell types, including parietal cells, macrophages, eosinophils, and dendritic cells. Elevated PD‐L1 on these cells competes with PD‐1 on T cells, inhibiting T‐cell‐mediated tumor destruction and thereby reducing the efficacy of anti‐PD‐1/PD‐L1 therapies [56].

### 4.3 Lactate Production by Bacteria and Its Impact on MSI

In the context of GC models, dysbiosis of the microbiota has been implicated in cancer progression through the production of metabolites and proinflammatory compounds, leading to the disruption of the normal physiological functions of epithelial cells [58]. Metabolic reprogramming occurs within the TME, resulting in elevated glucose consumption and the subsequent production of lactate. These metabolic changes are pivotal in processes such as angiogenesis, immune evasion, cell migration, and metastasis during carcinogenesis [59].

Infection with *H. pylori* facilitates the colonization of bacterial communities, with a notable presence of lactic acid bacteria (LAB), including *Streptococcus*, *Lactobacillus*, *Bifidobacterium*, and *Lactococcus*. LAB are potent inducers of ROS, which are capable of causing DNA damage. Additionally, they can reduce nitrates to nitrites, potentially leading to the accumulation of N‐nitroso compounds that favor the initiation of carcinogenesis [60].

The pathogenic mechanisms of *H. pylori* have been extensively studied. Advances in next‐generation technologies have enabled us to establish their role in contributing to the metabolic and biotic imbalance of the gastric epithelium. Novel molecular and epidemiological studies aimed at reinforcing early detection and eradication programs for *H. pylori* infection in asymptomatic children could significantly advance our understanding of microbiome–cancer relationships. These measures are expected to positively impact patient treatment [61, 62].

## 5 The Impact of Microbiota on Other Tumor Types

The study of the intratumoral microbiota in other cancer models has revealed several relationships between microorganisms and the process of carcinogenesis. To provide an overview of recent findings in the identification of microbial communities, the following table has been compiled (Table 1).

**TABLE 1:** Intratumoral microbiota in different models: Recent findings.

Type of cancer	Microorganisms	Microbiota changes	Effect on carcinogenesis	References
Oral squamous cell carcinoma (OSCC)	*Porphyromonas gingivalis* , *Tannerella forsythia* , *Treponema denticola* , *Fusobacterium*, *Dialister*, *Peptostreptococcus*, *Filifactor*, *Peptococcus*, *Catonella*, *Parvimonas*, and *Streptococcus anginosus*	Increase	Dysbiotic proinflammatory bacteriome Inhibition of DNA repair enzymes Caused double‐stranded DNA breaks	[63, 64, 65]
Pancreatic ductal adenocarcinoma (PDAC)	*Fusobacterium nucleatum* , *Porphyromonas gingivalis*	Increase	Cytokine secretion Tumor progression Secretion of neutrophilic chemokines and neutrophil elastase (NE)	[66, 67]
Lung cancer	*Acidovorax temperans* , *Haemophilus influenzae (NTHi)*, *Fusobacterium*	Increase	Associated with smoking and TP53 mutations Recruitment of neutrophils into inflamed tissues Resistance to immune checkpoint blockade (ICB)	[68, 69]
Breast cancer	*Borrelia burgdorferi* , *Prevotella copri* , *Escherichia coli* , *Staphylococcus epidermis*	Increase	Expression of inflammatory chemokines (CXCL8 and CXCL10) Altering the pattern of DNA methylation Caused double‐stranded DNA breaks	[70, 71, 72]
Prostate cancer	*Cutibacterium acnes*, *Clostridium scindens*	Increase	Promotes DNA double‐strand breaks Progression via the activation of AR signaling	[73, 74]
Ovarian cancer	*Acinetobacter seifertii*, *Peptostreptococcus*, *Bacteroides*, and *Prevotella*	Increase	Inhibit macrophage migration EOC progression through Hedgehog signaling	[75, 76]
Bladder cancer	*Syntrophobotulus*, *Granulicatella*, *Xanthomonas*, and *Pseudoalteromonas*, *Escherichia flexneri*	Increase	Changes in immune cell infiltration and cytokine profiles Metabolize BBN carcinogens	[77, 78]

## 6 Interventions Associated With the Microbiota in Antitumor Therapy

Microbiota has emerged as a critical factor in modulating cancer therapy outcomes, with distinct roles in both the TME and systemic immune responses. Recent studies have underscored the therapeutic potential of targeting microbiota to enhance the efficacy of cancer treatments through diverse mechanisms, including metal chelation, modulation of immune epitopes, and manipulation of gut microbial communities.

One promising approach involves metal chelation therapy, which has shown effectiveness in breast cancer treatment by depleting copper ions and elevating zinc ions. Copper, a key cofactor for various cellular processes, supports cancer progression by activating angiogenesis and enhancing the activity of copper‐dependent enzymes, such as lysyl oxidase, which facilitates extracellular matrix remodeling and tumor cell invasion. Chelation agents, such as tetrathiomolybdate, reduce intratumoral copper levels, suppress these pathways, and impede tumor growth and metastasis [79].

Notably, this therapy also reduces the levels of copper‐dependent bacteria within tumors, including *E. coli* and *F. nucleatum* , which can contribute to immune evasion by the tumor. Concurrently, an increase in zinc ions induces ROS, leading to apoptosis in cancer cells and amplifying antitumor effects. These advancements and the role of immune epitopes derived from intratumoral microbiota have gained attention [80].

MicroEpitope, an atlas cataloging microbial peptides associated with cancer, has demonstrated that specific bacterial antigens, such as those from *B. fragilis* and *F. nucleatum* , can elicit immune responses. These microbial peptides are present on major histocompatibility complex (MHC) molecules by antigen‐presenting cells, enabling the activation of T cells and promoting robust antitumor immunity. By priming the immune system to recognize and target tumor‐associated bacteria, these microbial epitopes contribute to improved immunogenicity within the TME and may serve as targets for developing microbiota‐driven immunotherapies [81].

In addition to the gut microbiota, it plays a crucial role in modulating systemic responses to cancer therapy. Certain gut bacterial species, including *Bifidobacterium longum* and *Lactobacillus acidophilus* , enhance the efficacy of ICIs by stimulating dendritic cell activity and increasing interferon‐gamma (IFN‐γ) production, which bolsters cytotoxic T‐cell responses against tumors [82].

Clinical strategies, such as FMT, are now being explored to modulate gut microbiota composition, especially in cases where resistance to ICIs has developed. FMT has shown potential in restoring therapeutic sensitivity, particularly in patients with melanoma who are unresponsive to ICIs. Moreover, the administration of prebiotics, probiotics, and postbiotics is under investigation for their potential to support gut microbial diversity, maintain intestinal barrier integrity, and optimize immune responses, although challenges in bioavailability and targeted delivery persist [83, 84].

Emerging from these studies is “cancer pharmacomicrobiomics,” which explores the interplay between the microbiome and the pharmacokinetics and pharmacodynamics of cancer therapies. This field aims to personalize cancer treatment by modulating the microbiota to enhance therapeutic efficacy and mitigate toxicity [85]. For example, gut microbial metabolites such as short‐chain fatty acids (SCFAs) have been implicated in modifying the TME, potentially enhancing the responsiveness of tumors to chemotherapy, radiotherapy, and immunotherapy. However, certain gram‐negative bacteria in the gut may induce immunosuppressive effects, leading to resistance to these therapies in some patients [86].

In summary, microbiota‐centered interventions, including metal chelation, immune epitope mapping, and gut microbiota modulation, represent a multifaceted strategy for enhancing cancer therapies. These approaches offer promising avenues for improving therapeutic efficacy and reducing adverse effects, positioning the microbiota as a pivotal factor in personalized cancer management. Further research is essential to unravel the molecular mechanisms underlying these interactions and develop effective, clinically applicable microbiota‐targeted therapies.

## 7 Conclusions

The interplay between cancer, MSI, and the microbiome reveals a multifaceted relationship that has profound implications for cancer therapy. Gut and intratumoral microbiomes are increasingly recognized as key modulators of both immune and therapeutic responses in patients with cancer. Microbial communities, particularly in the gut, influence drug metabolism, systemic immunity, and the TME, thereby affecting the efficacy and toxicity of treatments such as chemotherapy, radiotherapy, and immunotherapy.

The presence of specific bacterial species in the gut and TME can either bolster or hinder the effectiveness of therapies, as observed with ICIs. Dysbiosis, or an imbalance in microbial composition, has been consistently linked to poorer therapeutic outcomes, emphasizing the importance of a balanced microbiome in cancer treatment responses. MSI, characterized by a high mutational burden and neoantigen formation, has been established as a predictive biomarker for ICI response, particularly in CRC.

**FIGURE 4:**
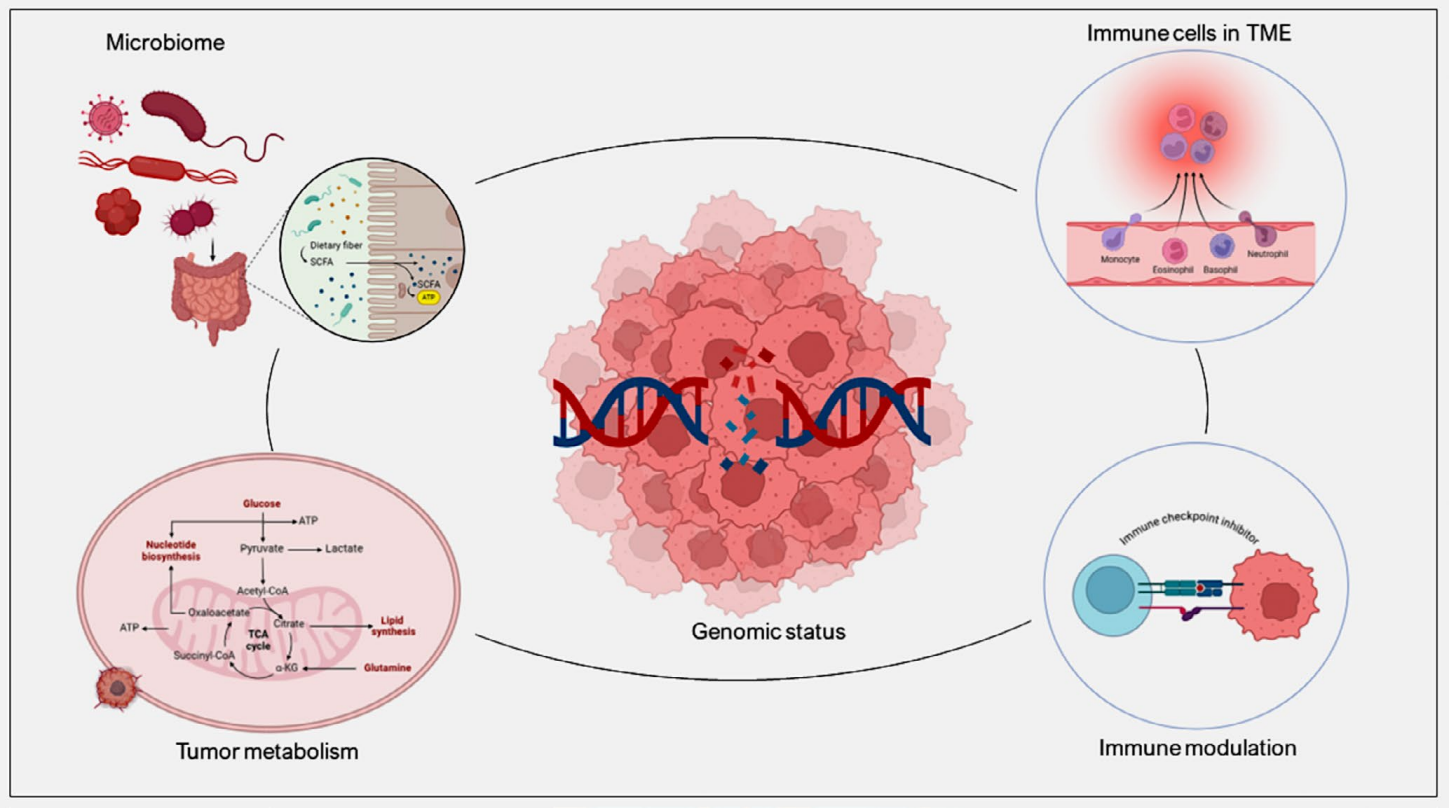
Schematic overview of various factors influencing cancer development and treatment response in the context of novel therapies and advanced technological tools.

MSI‐H tumors generally demonstrate enhanced immune activity, with elevated tumor‐infiltrating lymphocytes, which promotes sensitivity to ICIs. However, MSI‐H status alone does not universally predict immunotherapy outcomes across all cancer types, highlighting the complex interplay of genetic, microbial, and immunological factors that shape treatment responses (Figure 4).

## 8 Future Perspectives

Future research should focus on unraveling the mechanistic links between the microbiome and MSI status to enable precise cancer therapy. Specifically, studies should aim at the following:

*Identification of Microbial Signatures for Predictive Biomarkers*: Determining the specific microbial compositions associated with improved or diminished responses to cancer therapies could inform patient selection and personalized treatments.

*Development of Microbiome‐Targeted Therapies*: Interventions such as FMT, probiotics, and dietary modifications should be further explored for their potential to enhance treatment efficacy and mitigate adverse effects. These strategies could serve as adjuncts to conventional cancer therapies, particularly in MSI‐H tumors where immune modulation is pivotal.

*Understanding Tumor–Microbiome–Immune Interactions*: Given the role of intratumoral microbes in influencing immune cell infiltration and the immune landscape, further research into these dynamics will be crucial. Such insights could pave the way for microbiome‐based enhancements to immunotherapies, particularly in non‐CRC cancers where MSI‐H status alone does not reliably predict the therapeutic response.

*Incorporate Microbiome Modulation in Immunotherapy Protocols*: Integrating microbiome‐based strategies with ICI treatments holds promise for patients with MSI‐H cancers and may expand treatment benefits to other cancer types with lower ICI sensitivity.

In summary, the integration of microbiome modulation strategies into cancer therapy represents a promising approach. As we refine our understanding of MSI and the microbiome, these insights will drive the development of personalized, microbiome‐informed approaches to optimize cancer treatment outcomes and reduce resistance to therapies.

## Author Contributions

**Elizabeth Vargas‐Castellanos:** funding acquisition (lead), writing – review and editing (equal). **Andrés Rincón‐Riveros:** supervision (lead), writing – original draft (equal), writing – review and editing (equal).

## Conflicts of Interest

The authors declare no conflicts of interest.

## Data Availability

The authors have nothing to report.
